# Zinc blocks SOS-induced antibiotic resistance via inhibition of RecA in *Escherichia coli*

**DOI:** 10.1371/journal.pone.0178303

**Published:** 2017-05-22

**Authors:** Bryan E. Bunnell, Jillian F. Escobar, Kirsten L. Bair, Mark D. Sutton, John K. Crane

**Affiliations:** 1 Department of Medicine, Division of Infectious Diseases, University at Buffalo, Buffalo, NY, United States of America; 2 Department of Biochemistry, University at Buffalo, Buffalo, NY, United States of America; Centre National de la Recherche Scientifique, Aix-Marseille Université, FRANCE

## Abstract

Zinc inhibits the virulence of diarrheagenic *E*. *coli* by inducing the envelope stress response and inhibiting the SOS response. The SOS response is triggered by damage to bacterial DNA. In Shiga-toxigenic *E*. *coli*, the SOS response strongly induces the production of Shiga toxins (Stx) and of the bacteriophages that encode the Stx genes. In *E*. *coli*, induction of the SOS response is accompanied by a higher mutation rate, called the mutator response, caused by a shift to error-prone DNA polymerases when DNA damage is too severe to be repaired by canonical DNA polymerases. Since zinc inhibited the other aspects of the SOS response, we hypothesized that zinc would also inhibit the mutator response, also known as hypermutation. We explored various different experimental paradigms to induce hypermutation triggered by the SOS response, and found that hypermutation was induced not just by classical inducers such as mitomycin C and the quinolone antibiotics, but also by antiviral drugs such as zidovudine and anti-cancer drugs such as 5-fluorouracil, 6-mercaptopurine, and azacytidine. Zinc salts inhibited the SOS response and the hypermutator phenomenon in *E*. *coli* as well as in *Klebsiella pneumoniae*, and was more effective in inhibiting the SOS response than other metals. We then attempted to determine the mechanism by which zinc, applied externally in the medium, inhibits hypermutation. Our results show that zinc interferes with the actions of RecA, and protects LexA from RecA-mediated cleavage, an early step in initiation of the SOS response. The SOS response may play a role in the development of antibiotic resistance and the effect of zinc suggests ways to prevent it.

## Introduction

Our laboratories became interested in the *E*. *coli* SOS response to DNA damage because of role of the SOS responses in inducing Shiga toxin (Stx) from Shiga-toxigenic *E*. *coli* (STEC) [1, 2]. In addition to Stx production, the SOS response also triggers a myriad of bacterial cell responses, including DNA repair, elongation of bacterial cells, induction of error-prone DNA polymerases [3], induction of latent bacteriophage, and inhibition of cell division. Since Stx toxins are bacteriophage-encoded, it is not surprisingly that Stx1 and Stx2 are upregulated by the SOS response. Induction of error-prone DNA polymerases for trans-lesion synthesis of damaged DNA leads to an increased mutation rate, known as the mutator response or hypermutation. Our previous work showed that zinc inhibited not just Stx production, but also *recA* expression, elongation of bacterial cells, and production of live bacteriophage from STEC [4]. Since zinc inhibited the other aspects of the SOS response, we hypothesized that zinc would inhibit SOS-associated hypermutation as well.

The SOS response is strongly induced by treatments that damage bacterial DNA, including UV light, quinolone antibiotics, and mitomycin C. The SOS response is also induced by oxidant host defenses [5], which can be generated in the gastrointestinal lumen by xanthine oxidase (XO) from epithelial cells [6] as well as well as from polymorphonuclear neutrophils (PMNs) [7]. Kim et al. also recently reported that tetracycline antibiotics commonly used for growth promotion in cattle were also surprisingly strong inducers of Stx and of the SOS response in STEC [8], contrary to previous predictions [9].

Activation of the SOS response includes the expression of alternate DNA polymerases capable of trans-lesion synthesis of DNA. DNA polymerase IV, encoded by *dinB*, and DNA polymerase V, encoded by *umuDC*, are error-prone DNA polymerases induced by the SOS response [10–12].

Expression of *recA* RNA and RecA protein are early, measurable, and reliable indicators of the SOS response. We initially measured *recA* expression by quantitative RT-PCR in response to ciprofloxacin and mitomycin C in order to be able to optimize drug concentrations and time courses needed to observe maximal activation of the SOS, and to confirm that zinc blocked *recA* expression. Later, we also measured *recA* using a *recA-lacZ* reporter strain in a higher-throughput assay format that allowed us to test larger numbers of variables, antibiotics, and drugs. In addition to classical antibiotic inducers of SOS response, such as ciprofloxacin and mitomycin C, we also tested drugs such as 5-fluorouracil, zidovudine, and other antivirals and anti-cancer drugs, since these have been reported to induce the SOS response as well [13, 14], and demonstrated that zinc’s ability to inhibit the SOS response was not shared by most other transition metals.

## Materials and methods

### Materials

Mitomycin C, zinc pyrithione, methy-umbelliferyl-glucuronate (MUG), and the reagents used for the Miller assays for recA were purchased from Sigma-Aldrich (St. Louis, MO). Zidovudine, 5-fluorouracil, 5-azacytidine, paraquat, arsenic trioxide, and didanosine were also from Sigma-Aldrich. Ciprofloxacin was obtained from Bayer Pharmaceuticals. E-test strips were from Biomerieux (Durham, NC).

### Bacterial strains used

Bacterial strains used are listed in Table 1. Bacteria were grown overnight in LB broth at 37°C with 300 rpm shaking, then subcultured into the medium for the expression studies, usually DMEM medium. In this report, “DMEM” this refers to DMEM/F12 medium supplemented with 18 mM NaHCO_3_ and 25 mM HEPES, pH 7.4, but without serum or antibiotics. DMEM was used because it seems to accentuate the effects of zinc on bacteria, which is in part due to the phosphate in the medium [15]. Minimum inhibitory concentrations (MICs) of antibiotics were determined using E-test strips. For ciprofloxacin, we determined the MICs on DMEM agar, since the bacteria were exposed to ciprofloxacin in DMEM liquid, and for rifampin the MICs were determined on LB agar. LB plus rifampin plates were prepared fresh and used within 48 h since the antibacterial potency of the rifampin seemed to decrease with time, even though the plates were protected from light. For most strains, we used a concentration of rifampin equal to 2 times the MIC. Accordingly, for strain Popeye-1, we used LB + 10 μg/mL rifampin; for B171-8 we used LB + 8 μg/mL rifampin, and for EDL933 also 8 μg/mL rifampin. For Kpneu_707, however, we had to increase the concentration of rifampin to 2.8 X the MIC, or 45 μg/mL, to avoid uncountable lawns of growth.

**Table 1:** Bacterial strains used.

Type of Bacteria & Strain Name	Serotype, if relevant	Antibiotic MIC by E-test, μg/mL		Comments	References
		Ciprofloxacin, on DMEM	Rifampin, on LB		
STEC					
EDL933	O157:H7	0.023	4	Stx1, Stx2-producer	[16]
EDL933**R**	O157:H7	0.003	2	Δ*recA* mutant of EDL933; hypersusceptible to antibiotics	[17]
Popeye-1, TW14359	O157:H7	0.016	6	Stx2, Stx2c-producer; 2006 U.S. Spinach outbreak	[18]
TSA14	O26:H11	0.008	—	Stx1-producer	[19]
EPEC					
B171-8	O111:NM	0.012	4	classic human EPEC strain; Mexico	[20, 21]
ExPEc					
CP9		0.008	4	bacteremic isolate, NIH	[22]
Laboratory and Reporter Strains					
JLM281		0.012	10	*recA-lacZ* reporter strains	[23, 24]
*Klebsiella pneumoniae*					
Kpneu_707	not done	0.125	16	clinical isolate; bacteremic pneumonia isolate from a 73 year old man.	Erie County Medical Center Clinical Microbiology lab, July, 2007

Since bacteria are more sensitive to the DNA-damaging effects of mutagens when they are rapidly growing, we subcultured bacterial strains for 1 h prior to the addition of ciprofloxacin or zidovudine. Then the incubation was continued at 37° at 300 rpm shaking for 3 more hours before collecting bacteria for dilutions and plate counts. Dilutions were performed into 1X liquid Amies transport medium, allowing us the chance to go back and re-dilute and re-plate if our initial dilutions gave uncountable results.

### Assay for reversion to glucuronidase-positive

Classic STEC strains of the O157 serotype have lost their ability to utilize hydrolyze glucuronic acid from precursors as well losing their ability to use sorbitol. Studies of reversion to glucuronidase-positive in STEC O157 strains were done on custom-made selective fluorescence medium consisting of minimal medium (1X M9 salts, 2% casamino acids, 0.5 mM glucose, 4 mM sodium glucuronate, and 1.5 mM methy-umbelliferyl-ß-D-glucuronide hydrate (MUG, a fluorescent precursor that generates a fluorescent signal when cleaved by glucuronidase). The low concentration of glucose added, equivalent to 0.045% glucose, was intended to allow the mutagenized bacteria to survive long enough for mutations to become fixed, but not long enough to grow up to form visible colonies. The cultures and dilutions from the above experiments were also plated on LB agar in order to calculate the total number of cfu/mL to serve as the denominator in calculating the frequency of reversion to glucuronidase-positive. The fluorescent product generated from the cleavage of MUG (methyl-umbelliferone) diffuses out into the surrounding medium, making it difficult to count colonies if MUG + colonies are too densely crowded, a limitation of this method (see glucuronidase experiments below).

### Miller assay for expression of ß-galactosidase in bacterial reporter strains

Strain JLM281, the reporter strain containing the *recA-lacZ* construct, was used to measure *recA* expression in response to inducing antibiotics, zinc and other metals. JLM281 was a kind gift from Dr. Jay L. Mellies, Reed College, Portland, OR. We used a version of the Miller assay adapted to 96 well plates for higher throughput [25]. However, we used 0.1% hexadecyltrimethylammonium bromide (HTA-Br) detergent alone, without chloroform or sodium dodecyl sulfate (SDS), to permeabilize the bacteria. The buffers used are described in a Open WetWare website at: http://openwetware.org/wiki/Beta-Galactosidase_Assay_%28A_better_Miller%29.

We used the same conditions for the Miller assay as reported previously [4], and again omitted the addition of the NaCO_3_ STOP Buffer.

### *recA* RNA analysis by qRT-PCR

Bacterial RNA was collected into RNA Protect Bacteria Reagent (Qiagen, Valencia, CA). RNA was purified using GeneJet RNA purification kits from Thermo Fisher (Waltham, MA). Since *recA* RNA is of low abundance in the control, uninduced bacteria, it was necessary to use gene-specific primers for the reverse transcription step, which was carried out using kits from Invitrogen as previously described [18, 26]. PCR itself was carried out using SYBR Green detection using Master Mix from Bio-Rad or other supplies. PCR primers used were:

*recA* forward 5’-ggt aaa acc acg ctg acg tt-3’
*recA* reverse 5’ ata tcg acg ccc agt ttacg- 3’

As normalizing genes we again used primers for *rrsB* as described by Leverton & Kaper [27].

### Western immunoblotting

Bacterial whole-cell extracts were collected after 4 hours of subculture in DMEM. In order to reduce the viscosity of the extracts, which makes them hard to load accurately, we harvested bacteria by centrifugation at 13,000 *g* in an Eppendorf centrifuge, decanted the supernatants, and then resuspended in 100 μL of Tris-EDTA plus 30 μg/mL polymyxin B. Then 33 μL of 4X lithium dodecyl sulfate (LDS) sample buffer plus ß-mercaptoethanol was added. Antibody against LexA was rabbit polyclonal and was from Abcam, Cambridge, MA, and was used at the dilution of 1:12,000 suggested by the supplier. Electrophoresis was done with pre-cast 12% acrylamide Bis-Tris gels from Invitrogen. Proteins were transferred to nitrocellulose membranes using a traditional “wet” or tank transfer apparatus (Bio-Rad). The second antibody was goat anti-rabbit conjugated to horse radish peroxidase (HRP) from KPL (formerly K&P, Gaithersburg, MD), used at a dilution of 1:3000. Detection was by chemi-luminescence using reagents from a variety of suppliers, including KPL Laboratories and Abcam. After detection in the Bio-Rad Chemi-Doc MP imager, images files were saved and analyzed using Un-Scan-It Gel software for the MacIntosh computer (Silk Scientific, Orem, Utah).

### RecA-mediated LexA cleavage assays *in vitro*

In vitro LexA cleavage assays were carried out with purified RecA from New England Biolabs, Boston, MA, and purified LexA protein from Abcam. In general, we followed the instructions recommended in the on-line protocols from New England Biolabs. A 3X buffer concentrate was used so that after all additions the final buffer concentrations were 25 mM Tris-acetate, pH 7.8, plus 4 mM MgSO_4_. Dithiothreitol was added from a concentrated stock to yield 5 mM final. RecA was added to yield 0.2 μg per well, with LexA added to 0.16 μg per well, in a total volume of 20 μl using conical-bottomed 96-well plates. As the source of ssDNA needed to activate RecA, we used a 38-mer ReadyMade oligonucleotide, cDNA cloning primer, from Integrated DNA Technologies, Coralville, Iowa. The oligonucleotide was dissolved in Tris-EDTA buffer at a concentration of 8.6 μM, then diluted 20-fold to a final concentration of 0.43 μM in the assay. Zinc or other metals were added, and then the cleavage reaction was initiated by adding ATP or ATP-γ-S, and transferring the plate to a metal heater block in an incubator at 37°C for 15 min. Then 10 μL of water was added to each well, followed quickly by 10 μl of 4X LDS sample buffer plus ß-mercaptoethanol. For the experiments testing for auto-cleavage of LexA in the absence of RecA, we incubated LexA protein in 100 mM sodium borate buffer, pH 9, for 15 to 30 min at 37°C. Detection of LexA was by immunoblot as described above.

### Data analysis and statistics

Error bars shown on graphs and in Tables are standard deviations. Statistical signficance was tested by ANOVA using the Tukey-Kramer post-test for multiple comparisons, or by t-test when only 2 conditions were being compared. For bacterial counts, we used logarithmic transformation of the raw data, as recently recommended for this type of microbiological data [28], since bacterial titers often have a skewed distribution. In some of the LexA immunoblots, in order to combine data from different experiments and blotted on different days, we expressed the LexA band densities as a percent of the control lacking the adenine nucleotide.

## Results

**Fig 1:**
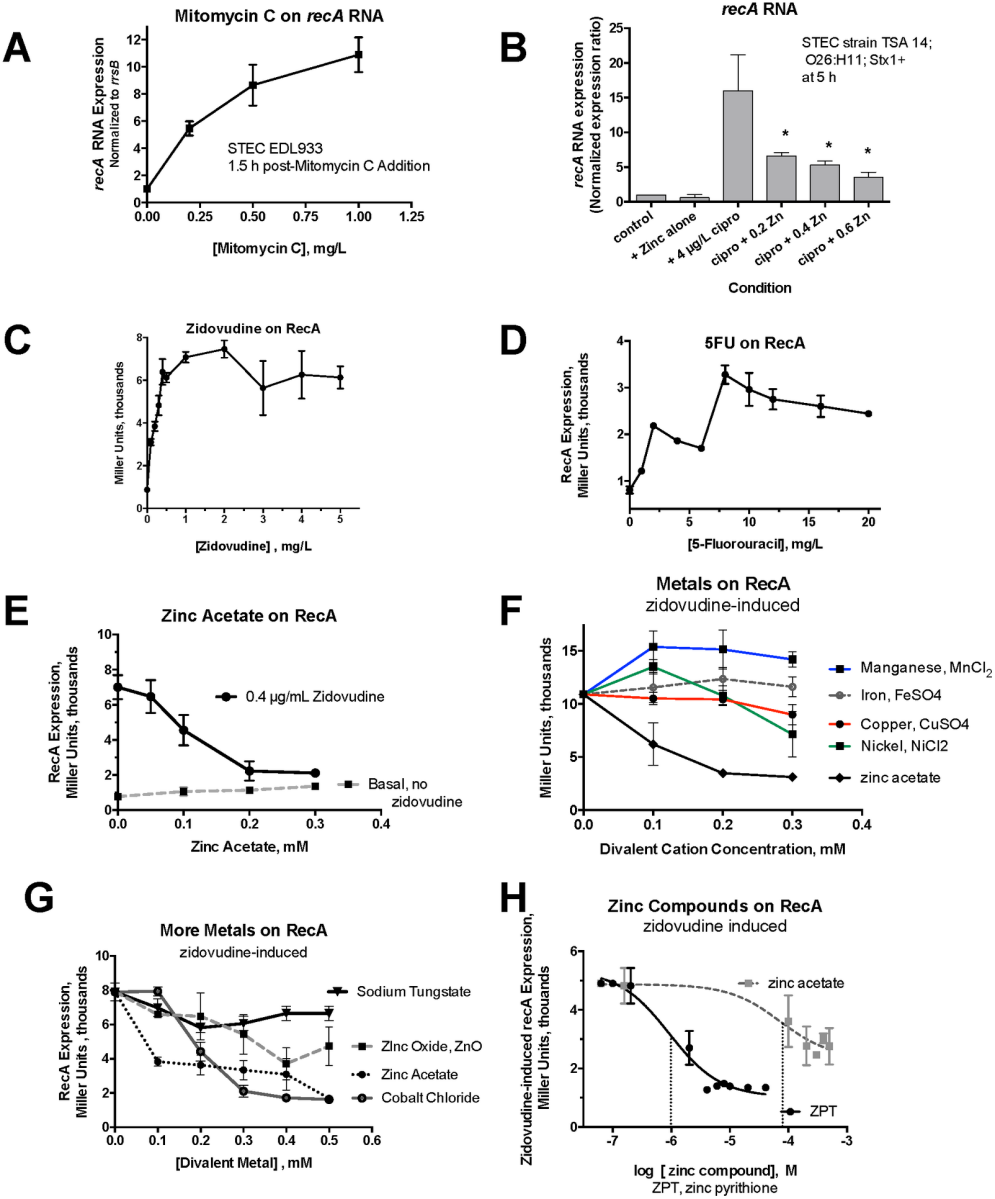
Activators and inhibitors of the SOS Response in E. coli. Panels A and B, induction and inhibition of *recA* in STEC strains by qRT-PCR. Panel A, effect of mitomycin C on *recA* expression in STEC EDL933. Panel B, effects of ciprofloxacin and zinc acetate on *recA* expression in STEC TSA14. Panels C-H, effects of inducers and inhibitors of *recA* as measured using the Miller Assay and reporter strain JLM281. For all panels the activators and inhibitors were added 1 h after beginning the subculture in DMEM broth. *, significant by ANOVA compared to ciprofloxacin alone. In panels E-F the inhibitory effect of zinc on zidovudine-induced *recA* was significant for 0.2 mM and higher, despite the lack of asterisks. Panel H, the vertical dotted lines represent the IC_50_ of zinc pyrithione (ZPT) and zinc acetate, and show that zinc pyrithione was 89 times more potent than zinc acetate in inhibition of zidovudine-induced *recA*.

Fig 1 shows experiments done to optimize the activation and inhibition of the SOS response. As mentioned above, *recA* expression is an early, reliable, and quantifiable marker of the onset of the SOS response. Fig 1A shows that the abundance of *recA* RNA increases markedly in response to mitomycin C in STEC strain EDL933, in agreement with previous work [29]. Fig 1B shows that ciprofloxacin also strongly induced *recA* RNA expression in a different STEC strain, TSA14. Ciprofloxacin-induced *recA* expression was inhibited by zinc acetate, as we expected based on the known inhibitory effect of zinc on Stx toxin production. Fig 1C shows that *recA* expression was also measurable using a *recA-lacZ* reporter strain, JLM281. Like mitomycin C and ciprofloxacin, zidovudine also strongly induced *recA*. Fig 1D shows that 5-fluorouracil, also a pyrimidine analog, induced recA expression, although not as powerfully as zidovudine. Zinc acetate inhibited zidovudine-induced *recA* expression (Fig 1E) with an inhibitory potency similar to its effects against ciprofloxacin-induced *recA* in Fig 1B. Fig 1F and 1G compare the ability of zinc to inhibit zidovudine-induced *recA* expression with that of other divalent metals. Fig 1F shows that zinc was more effective in inhibiting *recA* expression than manganese, iron, copper, or nickel. Fig 1G shows that zinc acetate was more effective than zinc oxide in inhibition of *recA*, while sodium tungstate had no effect. Cobalt chloride did inhibit zidovudine-induced *recA* expression and was slightly more potent than zinc acetate in this regard (Fig 1G). Cobalt is 160 times more toxic than zinc, however, based on the inverse ratios of the tolerable upper limits for human consumption, so this might limit or rule out its use in *in vivo* experiments. Fig 1H shows that the ionophore zinc pyrithione was 72-fold (1.8 logs) more potent than zinc acetate in its ability to inhibit *recA* expression in the reporter strain. Zinc pyrithione has been studied mostly for its antifungal properties and very little studied with regard to its possible effects on bacterial pathogenesis.

**Fig 2:**
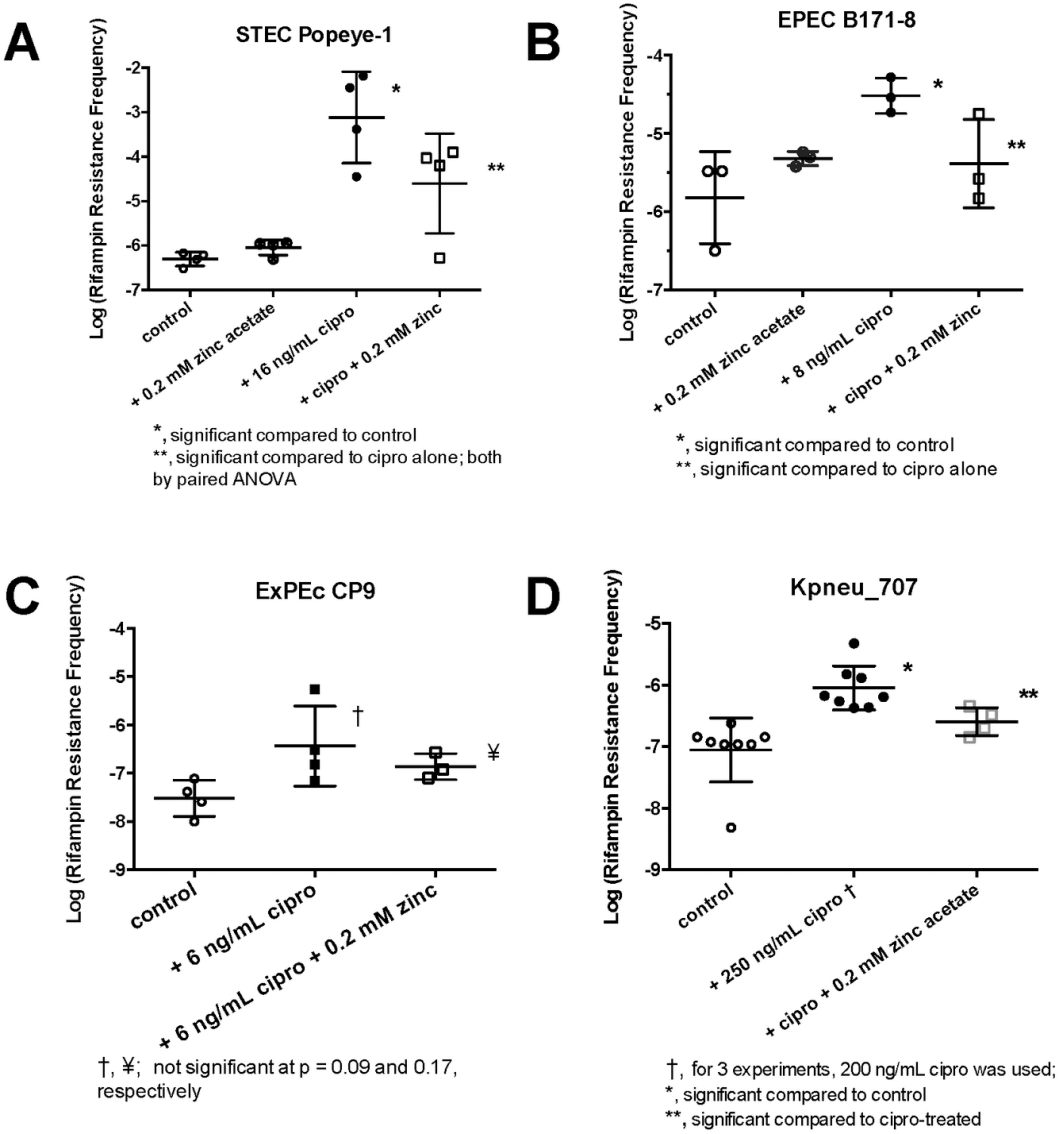
Induction and inhibition of hypermutation by zinc in various strains of bacteria. For each panel, the bacterial strain indicated was treated for 3 hours with and without the concentration of ciprofloxacin indicated, as guided by the ciprofloxacin MIC for each strain, and ± 0.2 mM zinc acetate. Then serial dilutions were performed, and plated on plain LB agar to determine the total number of bacteria, and on LB + rifampin, to determine the number of rifampin resistant colonies per mL. The rifampin resistance frequency was calculated for each condition. The rifampin concentrations, for each strain were, in μg/mL: Popeye-1, 12; B171-8, 8; CP9, 8; and Kpneu_707, 45. Paired ANOVA was by Kruskal- Wallis for non-parametric data due to skewing.

The detailed dose response relationships in Fig 1, both for the activators of the SOS response and the inhibitors, were next used to optimize conditions in which to test for hypermutation in subsequent experiments. Fig 2 shows the results of experiments in which we sought to determine if hypermutation could be induced in *E*. *coli* strains. We began with STEC strains because we knew how to strongly induce the SOS response due to our previous work on Stx toxins. If SOS-induced mutation was observed, then we did additional experiments to test if the mutator response could be reversed by zinc acetate. Following that, we tested other pathotypes of E. coli, including enteropathogenic strains (EPEC), extra-intestinal pathogenic E. coli (ExPEc), and a *Klebsiella pneumoniae* strain. As an initial experimental plan, we sought to see if exposure to an SOS-inducing agent could induce hypermutation to rifampin, also known as rifampicin. Rifampin, an RNA polymerase inhibitor, is a large molecule that requires multiple points of contact with the enzyme to achieve inhibition. Rifampin resistance can arise from mutations resulting in amino acid substitutions at 24 different amino acid residues in RNA polymerase, most of them in 3 different clusters within the ß-subunit of RNA polymerase [30–32], giving *E*. *coli* multiple routes by which to achieve rifampin resistance. In addition, rifampin resistance can emerge due to mutations in other loci, such as the AcrAB efflux pathway; [33, 34]. We used rifampin at concentrations 2 to 3 times above the MIC, which gave sufficiently a low background of rifampin resistance against which to measure hypermutation, but for strain JLM281 we increased the rifampin concentration to 100 mg/L, or 10 times the MIC. We usually used ciprofloxacin at a concentration 1/3 of the MIC, although we varied this concentration from ¼ of the MIC to ½ of the MIC if needed based on pilot experiments.

Fig 2A shows that a 3 h exposure to ciprofloxacin strongly increased the rifampin resistance frequency in STEC Popeye-1, by ~ 3 logs, to a level ~ 1000-fold above control. Zinc acetate alone had minimal effect on mutation rates in the absence of ciprofloxacin, but 0.2 mM zinc reversed the effects of ciprofloxacin. Zinc acetate strongly reduced cipro-induced hypermutation, a 32-fold compared to ciprofloxacin alone, back toward the control frequency of rifampin resistance.

Fig 2B shows that ciprofloxacin also induced hypermutation in EPEC B171-8, a classic human EPEC strain. In B171-8, ciprofloxacin triggered a 1.3-log (20-fold increase) in the rifampin resistance frequency, while 0.2 mM zinc again reduced ciprofloxacin-induced rifampin resistance significantly, a mean of a 0.9 log decrease (8-fold decrease).

Fig 2C shows the results we obtained when we subjected ExPEc strain CP9 to the same mutation-inducing protocol. In CP9, the spontaneous mutation rate appeared lower than in the STEC and EPEC strains, and CP9 was less susceptible to induction of hypermutation by ciprofloxacin. Although the effects of ciprofloxacin and zinc did not achieve statistical significance in Fig 2C, the trends seen paralled those seen in the other *E*. *coli* strains. Results similar to those in Fig 2C were also observed with other ExPEc strains, including fresh clinical isolates (data not shown for the latter).

Fig 2D shows the results obtained when we sought to extend our results to *Klebsiella pneumoniae*. With *K*. *pneumoniae*, we were unable to observe SOS-induced mutation response using ciprofloxacin concentrations below the MIC (preliminary experiments not shown). However, when we increased the concentration of ciprofloxacin to twice the MIC, we did observe an increase in the rifampin resistance frequency, an approximate 11-fold increase compared to control, and again zinc acetate reduced the ciprofloxacin–induced rifampin resistance by 4-fold compared to ciprofloxacin alone. In additional experiments with laboratory strain JLM281, we were able to observe the hypermutation response even when we used a rifampin concentration of 100 mg/L, which was 10 X the rifampin MIC for this strain (S1 Fig). Ciprofloxacin–induced hypermutation was fully blocked by zinc acetate in this experimental variation was well (data not shown for JLM281). We have also extended the findings in Fig 2 to *Enterobacter cloacae*, verifying again that ciprofloxacin can induce hypermutation and that the mutator response is inhibited by zinc (data not shown for Enterobacter). In additional control experiments, we found that zinc did not have significant effects on the susceptibility of the strains tested to the antibiotics used, as measured by the MIC values to ciprofloxacin and rifampin (S2 Fig); such effects, if they had been observed, might have confounded the results we observed in Fig 2.

In Table 2 we extended our studies of SOS-induced mutation to STEC strain EDL933 and its Δ*recA* mutant, EDL933R. Since Δ*recA* mutants are hypersusceptible to antibiotics, in these studies we had to adjust our approach by using ciprofloxacin concentrations equivalent to 1/3 of the MIC and used rifampin concentrations at 2 times the MIC for each strain; the concentrations were lower for EDL933R as shown in Table 2. The top portion of Table 2 shows the results of 5 experiments with the wild-type EDL933, in which ciprofloxacin induced an increase in rifampin resistance frequency, with a mean increase of 20-fold compared to control cultures. In 2 of the 5 experiments, a ciprofloxacin + zinc condition was included, and zinc reduced the ciprofloxacin-induced increase a mean of 27- fold.

**Table 2:** Effect of Δ*recA* mutation on ciprofloxacin-induced hypermutation in STEC EDL933.

Experiment Number	Strain	Rifampin Resistance Frequency per million				Fold-Change in Rifampin- Resistance Frequency with cipro, Mean ± SD	Fold-Decrease in Rifampin Resistance with Zinc compared to cipro alone Mean ± SD
		Control	Cipro Conc Used *	Cipro-Induced Rif Resistance	+ Cipro + 0.2 mM zinc		
1	EDL933 (wt)	0.05 ± 0.01	6 ng/ml	1.48 ± 0.36	not done	29.5-fold increase	not done
2	EDL933	0.141 ± 0.05	6 ng/ml	0.625 ± 0.17	not done	4.3-fold increase	not done
3	EDL933	0.063 ± 0.01	7 ng/ml	0.544 ±0.33	0.102 ± 0.1	8.6-fold increase	5.3 -fold
4	EDL933	0.116 ± 0.05	7 ng/ml	4.29	0.06 ± 0.05	37-fold increase	71-fold
5	EDL933	0.256 ± .038	8 ng/ml	5.4 ± 1.5	not done	21.1-fold increase	not done
						**20.1 ± 13.7-fold increase with cipro**	**27.2** ± 37-fold decrease due to zinc

			Cipro Conc Used **				
1	EDL933R ΔrecA	0.54 ± 0.11	0.7 ng/mL	0.25 ± 0.19	not done	2.1-fold decrease	N/A
2	EDL933R	2.00 ± 0.27	1 ng/ml	1.53 ± 0.43	not done	1.3- decrease	N/A
3	EDL933R	11.2 ± 3.2	1.2 ng/ml	4.17 ± 1.62	not done	2.7-fold decrease	N/A
						**Mean 2.0 ± 0.7 fold decrease with cipro**	N/A

*, As shown in Table 1, the ciprofloxacin MIC of the wild-type EDL933 is 23 ng/mL

**, Ciprofloxacin MIC for EDL933R mutant is 3 ng/mL.

For both strains we used concentrations of ciprofloxacin bracketing the value of one-third of the MIC. The concentration of rifampin in the LB plates used for detection of rifampin resistance was 10 μg/mL for the wild-type and 4 μg/mL for the EDL933R mutant.

In the lower portion of Table 2, ciprofloxacin was never able to increase the rifampin resistance frequency in the EDL933R mutant. In fact, in 3 of 3 experiments ciprofloxacin treatment triggered a small paradoxical decrease in the rifampin resistance frequency. The lack of any increase in rifampin resistance in EDL933R following ciprofloxacin treatment confirms with the critical role of *recA* in the SOS response and in hypermutation in *E*. *coli*.

In order to move beyond rifampin resistance as the only measure of hypermutation, we considered whether mutagenic stimuli would be able to trigger a reversion in the ability to hydrolyze glucuronic acid from precursors in STEC strains. Classic STEC strains in the O157 serotype have a 2-nucleotide GG insertion upstream of the ß-glucuronidase gene, resulting in a loss of that enzyme [35]. Along with their well-known inability to use sorbitol, and this inability to hydrolyze glucuronic acid from precursors has been used as the basis for selective indicator agars for O157 bacteria, such as MUG MacConkey agar (Sigma-Aldrich) and Chromagar O157 (Chromagar, Paris, France). We hypothesized that induction of error-prone DNA polymerases during the SOS response might result in frame-shift mutations that would restore the reading frame for the ß-glucuronidase gene in classic STEC. We began our experiments using MUG MacConkey agar, but found that glucuronidase-positive colonies were not very bright, possibly due to the neutral red dye in that medium. We therefore designed a custom minimal medium agar containing sodium glucuronate, the fluorescent substrate methyl-umbelliferyl-ß-D-glucuronide, and a very low (0.5 mM = 0.045%) concentration of glucose. We tested whether ciprofloxacin or zidovudine could also cause an increase in the number of fluorescent colonies in STEC strain Popeye-1.

**Fig 3:**
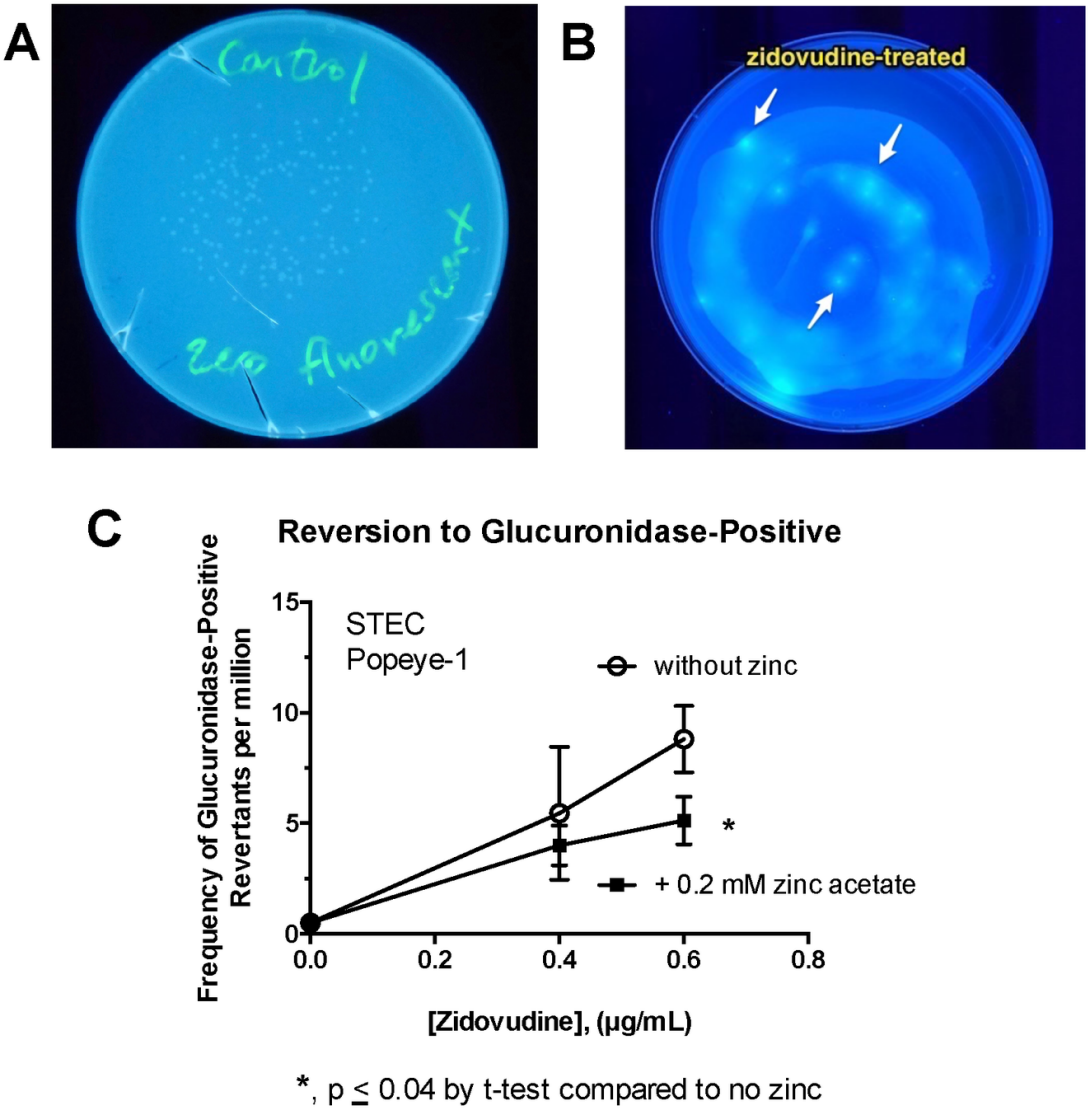
Assaying for hypermutation using ß-glucuronidase assay on MUG Selective agar. MUG selective agar was formulated using methyl-umbelliferyl-glucuronide (MUG) as stated in Materials and Methods. STEC Popeye-1 was treated with zidovudine as indicated for 3 h, followed by serial dilutions and plating on MUG Selective agar as well as on plain LB plates to determine total counts. Panel A, plate of untreated Popeye-1 on MUG agar, showing that although faint colonies are visible they do not fluoresce. Panel B, a heavy inoculum of zidovudine-treated Popeye-1 formed a lawn of growth, within which several brightly fluorescent colonies are visible. Panel C, dose-response of zidovudine on the frequency of glucuronidase-positive colonies, in the absence and presence of zinc.

Fig 3A and 3B show the appearance of STEC Popeye-1 in the absence (3A) and presence (3B) of zidovudine treatment. In Fig 3B, a minority of fluorescent colonies are visible within a lawn of Popeye-1 bacteria. Fig 3C shows that exposure to zidovudine for 3 h triggered a dose-dependent increase in the frequency of fluorescent colonies (a few of which are indicated by arrows) on the MUG Selective Medium. The effect of zidovudine was again inhibited by 0.2 mM zinc acetate. Reversion to glucuronate-positive phenotype was also observed with ciprofloxacin using this method (data not shown). One limitation of the MUG agar method is that colonies can be difficult to count if they are too numerous, due to diffusion of the fluorescent dye out of the colony into the surrounding medium (see Fig 3B). This limitation can be overcome by proper dilutions, however. A strength of this method is that small numbers of revertants can be seen against a heavy background, making this method an option for rapid screening. More importantly, the experiments with MUG agar in Fig 3 showed that the zidovudine-induced and ciprofloxacin-induced hypermutation phenomenon could be observed using an assay without rifampin or any other antibiotic selection.

**Fig 4:**
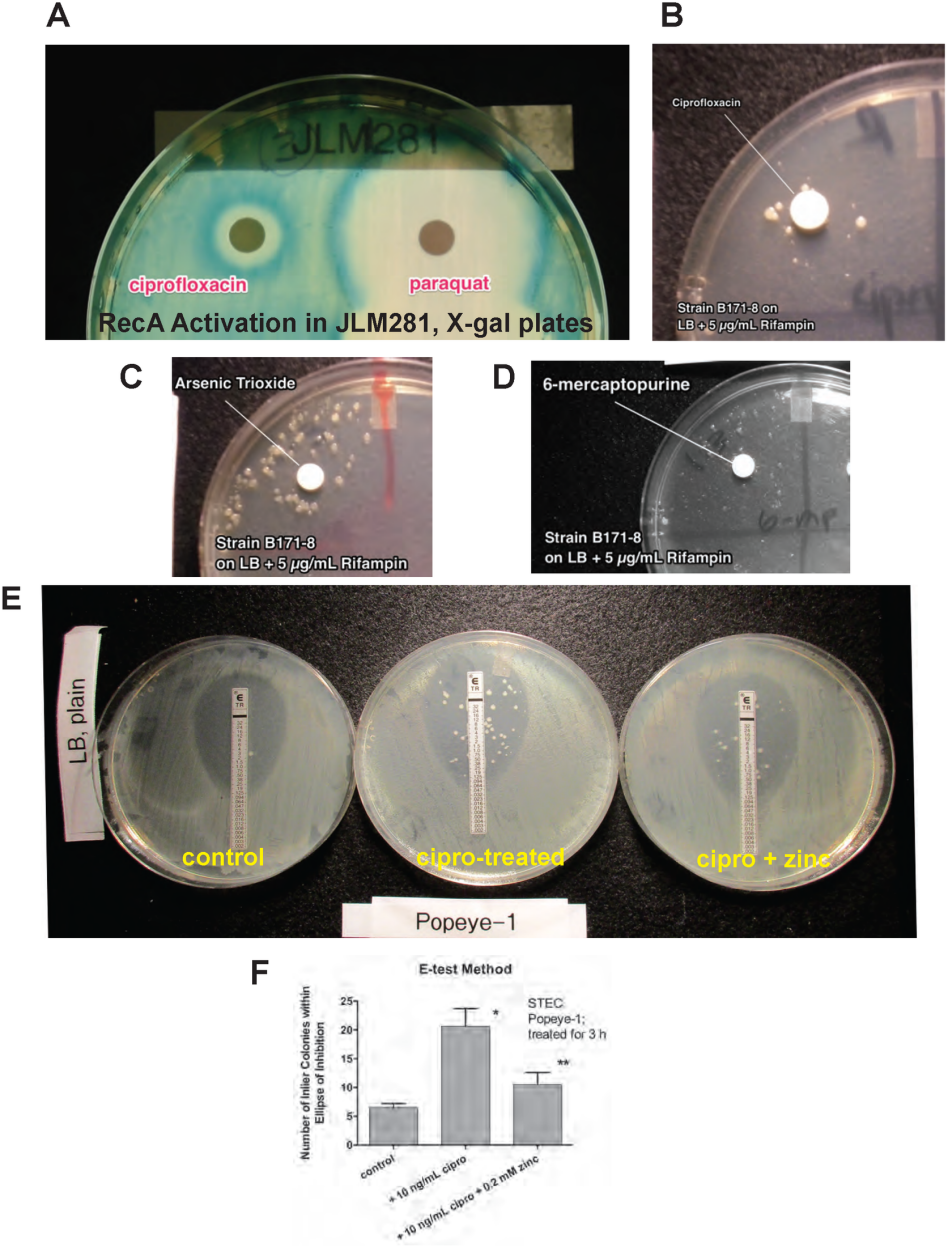
Attempts to develop more rapid screening methods for hypermutation in *E*. *coli*. Panels A and B, using *recA-lacZ* reporter strain JLM281 on plates containing LB + 150 μg/mL X-gal. Panel A, compared to ciprofloxacin (positive control), the herbicide paraquat also induced *recA* expression; 10 μl of a 50 mg/mL paraquat solution was spotted onto a sterile blank test disk. Panels B- D, testing for hypermutation in EPEC strain B171-8 on LB + 5 μg/mL rifampin using antibiotic test disks. Plates were inoculated with a 1:5 dilution of an overnight culture of B171-8 using a sterile cotton swab and a criss-cross pattern over the entire plate. Panel B, 10 μl of 1 μg/mL ciprofloxacin was spotted on the disk. A ring of rifampin-resistant colonies grew up in the vicinity of the ciprofloxacin. Panel C, same as Panel B, except that 10 μL of 40 mM arsenic trioxide was spotted onto the blank disk. Panel D, same as Panels B and C, but using 10 mM 6-mercaptopurine. Panels E and F, attempt to create a semi-quantitative screening method for hypermutation. STEC Popeye-1 was grown in the absence or presence of 10 ng/mL ciprofloxacin ± 0.2 mM zinc acetate, then diluted into sterile saline to achieve an OD_600_ of 0.2 for each culture. The diluted cultures were spread using a sterile cotton swab and a criss-cross pattern to cover the entire plate, then a trimethoprim E-test strip was applied to each plate. Each condition was plated in triplicate. Panel E, the ciprofloxacin-treated cultures (middle Petri dish) showed many more inlier colonies within trimethoprim’s zone of inhibition than in the control (left) or the ciprofloxacin + zinc condition (right). Panel F, the number of inlier colonies were counted in triplicate for each condition and are shown. *, significantly more than control; **, significantly fewer than ciprofloxacin alone, both by ANOVA.

Since the quantitative assays in Figs 2 and 3 and Table 2 were quite labor intensive, we sought more rapid methods that might be used to identify drugs capable of inducing the mutator response, and antibiotics against which resistance could be induced. Fig 4 shows some of the results of these more rapid, but non-quantitative or semi-quantitative assays. Fig 4A shows that agar plates made with X-gal and spread with the reporter strain JLM281 could be used to screen for non-traditional compounds capable of inducing *recA* expression. Fig 4A shows that the herbicide paraquat strongly induced the SOS response, with ciprofloxacin included as a positive control. Other agents that induced *recA* in this assay included didanosine (ddI, an anti-retroviral), 5-azacytidine, and arsenic trioxide. We also sought to determine if we could detect induced hypermutation on LB agar + rifampin agar without the pre-exposure to inducing agents in liquid broth cultures used in Figs 2 and 3. Fig 4, panels B-D, show experiments done with EPEC strain B171-8 on LB + 5 μg/mL rifampin, revealing that ciprofloxacin, arsenic trioxide, and 6-mercaptopurine all induced the growth of rifampin-resistant colonies in a ring or halo around the inducing drug. Arsenic was tested because oxides of arsenic are not only genotoxic [36] but also induce oxidant stress on bacteria [37], and oxidant and nutritional stress seem to prolong and intensify the SOS response [38]. Fig 4E shows the result of a 3 hour exposure to ciprofloxacin on the subsequent response of STEC Popeye-1 to trimethoprim, where trimethoprim was supplied the form of an E-test strip. As seen in Fig 4E, exposure to ciprofloxacin did not increase the trimethoprim MIC, which remained at ~ 0.125 μg/mL in the treated and untreated cultures. Ciprofloxacin exposure did, however, more than triple the number of resistant “inlier” colonies that appeared within the ellipse of inhibition. Fig 4F shows a graph quantifying the effects seen in Fig 4E, with each plate done in triplicate. Again, the effects of ciprofloxacin and zinc were both significant in this assay. The results of Fig 4, Panels E and F, suggest that the hypermutation phenomenon could be screened for on solid media using E-test strips or antibiotic disks. In addition, trimethoprim should be added to the list of antibiotics to which resistance can be induced by ciprofloxacin.

Table 3 summarizes the results of the SOS and hypermutation studies shown so far, emphasizing that there is a longer list of drugs and chemicals capable of inducing the SOS response, and a shorter list of drugs for which hypermutation has actually been demonstrated. If we include other drugs and treatments reported in the literature to activate the SOS response, the number of agents potentially able to trigger hypermutation phenomenon would be much longer [1, 8, 39].

**Table 3:** Summary of agents capable of inducing hypermutation and types of antibiotic resistance induced.

Drugs and Chemicals that can Induce the SOS Response, by *recA* Assays	Drugs and Chemicals that can Induce Hypermutation			Drugs to Which Resistance Can be Induced
		Quantitative	Qualitative	
ciprofloxacin	ciprofloxacin	√	√	rifampin, trimethoprim, chloramphenicol
Pyrimidine analogs				
zidovudine (AZT)	zidovudine	√		rifampin, chloramphenicol
5-fluorouracil				
5-azacytidine				
Purine analogs				
6-mercaptopurine	6-mercaptopurine		√	rifampin
didanosine, ddI				
arsenic trioxide, As_2_O_3_	arsenic trioxide		√	rifampin, chloramphenicol
paraquat	paraquat		√	rifampin

We next carried out experiments to try to determine the mechanism of action of zinc in inhibiting hypermutation.

**Fig 5:**
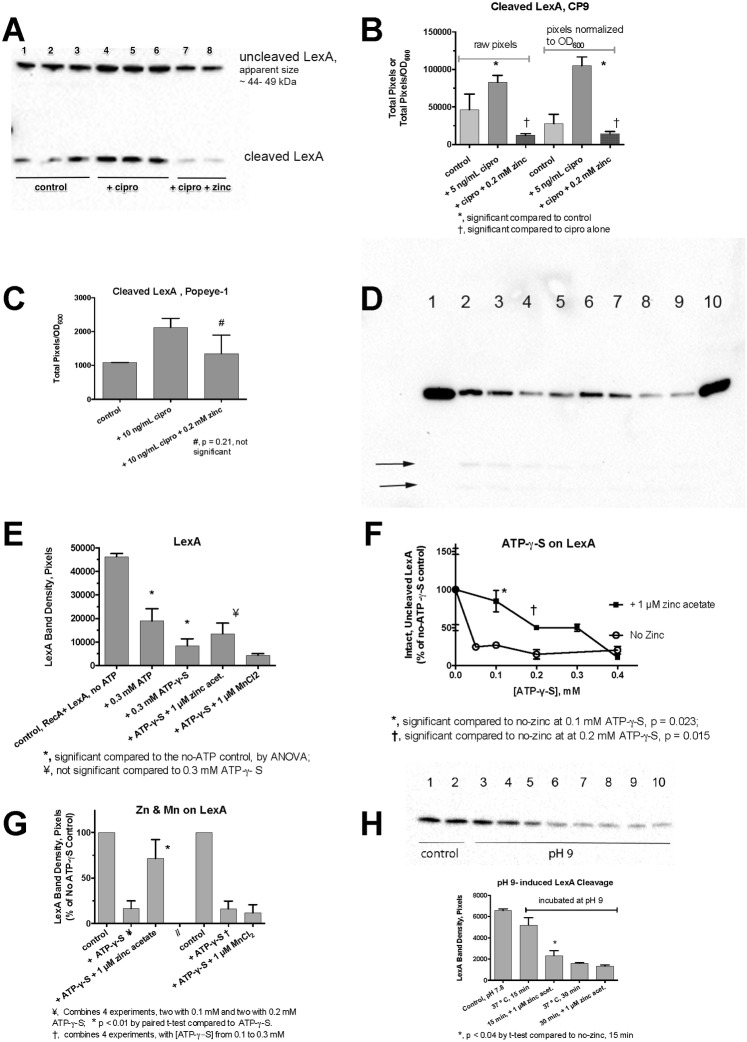
Regulation LexA by SOS activators and by zinc in *E*. *coli*. Panel A, immunoblot for LexA in whole-cell extracts of cultures of *E*. *coli* CP9 after a 3 h exposure to ciprofloxacin with and without zinc. Uncleaved LexA appeared to migrate in the form of a LexA dimer in these blots; while the cleaved LexA product ran at ~ 15 kDa. Panel B, densitometry scan of blot in Panel A, raw (left) and corrected for the effects of treatment on growth (right panel). Panel C, densitometry scan of a LexA blot (not shown) after a 1 h exposure to ciprofloxacin in Popeye-1. Panels D- H, RecA-mediated LexA cleavage assays in vitro, showing immunoblots against LexA. Purified LexA and RecA were incubated in vitro in the presence of absence of necessary cofactors, such as ssDNA and ATP or ATP- γ -S as described in the Methods section Panel D, RecA-mediated cleavage of LexA. An unlabeled lane to the left of lane 1 contained RecA alone, showing that the antibody does not cross-react between the two proteins. All the labeled lanes in Panel D received RecA, LexA, and a 38-mer oligonucleotide. Lane 1, no ATP; Lanes 2 and 3 also received 0.3 mM ATP; Lanes 4 and 5 also received 0.3 mM ATP-**γ-**S. Faint LexA cleavage products were visible in lanes 2–5 in the original blots, ***arrows***; Lanes 6 and 7, plus ATP-**γ-**S and 1 μM zinc acetate; Lanes 8 and 9, plus ATP-**γ-S** and 1 μM MnCl_2_; Lane 10 received 0.3 mM GTP, which does not support RecA activation, as an additional control. Panel E, densitometry scan of the chemiluminescence signal from the blot shown in Panel D. Panel F, dose-response relationship of ATP-**γ-**S concentration vs. LexA cleavage in the absence and presence of 1 μM zinc acetate, showing protection by zinc against LexA cleavage at 0.1 to 0.3 mM ATP-γ**-**S. Panel G, combined results of 4 separate experiments testing for the effect of zinc acetate, and four experiments with MnCl_2_ on LexA cleavage, with results normalized to the no- ATP-**γ-**S control so that separate experiments could be compared. Panel H, lack of protection by zinc on LexA auto-cleavage induced by incubation at pH 9. Control lanes 1 and 2 show LexA kept at pH 7.8; Lanes 3–6 show LexA protein incubated for 15 min at pH 9, 37°. Lanes 7–10 show samples incubated at pH 9 for 30 min, 37°. Lanes 5–6 and 9–10 also received 1 μM zinc acetate.

In *E*. *coli* bacterial culture, addition of ciprofloxacin and zinc had strong effects on the cleavage of the LexA protein, as detected by Western immunoblot. Fig 5A shows that ciprofloxacin treatment increased the amount of cleaved LexA, and 0.2 mM zinc blocked the appearance of the cleaved form in *E*. *coli* CP9. Interestingly, the increase in the amount of cleaved LexA was not accompanied by a diminution in the amount of uncleaved LexA observed (lanes 4–6, top band). This suggests re-synthesis of LexA by this point in the SOS response, after 3 h of treatment, as previously observed [40]. Trends similar to that observed in CP9 were also observed in STEC Popeye-1, but in the STEC strain the intact LexA bands were fainter, and disappeared more quickly after ciprofloxacin treatment (Blots not shown). Fig 5B shows the results of densitometry scans of the LexA blot from Fig 5A, showing that the effects of ciprofloxacin and zinc were statistically significant. In STEC Popeye-1, the LexA bands showed a similar trend, but the inhibitory effect of zinc did not achieve statistical significance (Fig 5C). Nevertheless, the results of Fig 5, Panels A-C indicated that zinc was having an effect early in the SOS response, and blocking the cleavage of the LexA repressor. To investigate this further, we conducted LexA cleavage assays in vitro using purified RecA and LexA proteins [41].

Fig 5D shows that, in the presence of ATP or its hydrolysis-resistant analog ATP-**γ-**S, RecA induced the cleavage of LexA in vitro (lanes 2–5). Addition of 1 μM zinc acetate inhibited LexA cleavage (lanes 6 and 7), but 1 μM MnCl_2_ did not. Unlike ATP, GTP did not serve as a co-factor for the cleavage of LexA (lane 10). Faint LexA cleavage products could be seen in some lanes (see 2 lower arrows indicting bands in lanes 2–5), but these were too faint for accurate quantitation, so we focused on quantitation of the intact, uncleaved LexA. Fig 5E shows the results of the densitometry scan of the blot shown in Fig 5D. As part of experiments done to optimize reaction conditions, we noted interesting results on the protective effect of zinc in the presence of varying concentrations of ATP-γ-S. While ATP-γ-S was needed to observe LexA cleavage, we noted that at higher concentrations of ATP-γ-S the protective effect of zinc diminished and disappeared (Fig 5F). This suggests that zinc may be perturbing the conformation of RecA in such a way that reduces its ability to bind ATP or ATP-**γ-**S, and thereby preventing its activation. Although the protective effect of zinc in Fig 5E failed to reach statistical significance, when we combined the results of 4 separate experiments, zinc did have a significant effect (Fig 5G). Manganese, however, failed to show any protection, and in 3 of 4 experiments actually decreased the amount of intact LexA remaining (right side of Fig 5G). In other preliminary experiments not shown, 1 μM CuSO_4_ and FeSO_4_ also did not protect against LexA cleavage. The protection by zinc, but not by other metals, echoes the results observed in assays of *recA* induction in Fig 1F.

As an additional control, we tested if zinc had any effect on RecA-independent cleavage of LexA. At pH 9, the auto-protease activity of LexA becomes active without the need for RecA, ssDNA, or ATP. The blot in the top portion of Fig 5H shows the auto-cleavage of LexA. Zinc acetate at 1 μM showed no protection against pH 9-induced LexA cleavage, and instead seemed to accelerate the auto-cleavage at the earlier time point. In summary, the results of Fig 5 show that zinc blocks the onset of the SOS response by preventing cleavage of the LexA repressor. The results of Fig 5F and 5H would point to RecA rather than LexA as the target of zinc action.

## Discussion

Our interest in the SOS response began because of the role of this important stress response in the regulation of Stx toxin production in STEC [1]. As we began to appreciate that zinc inhibited the SOS response [4], we hypothesized that zinc’s effects might extend well beyond STEC and Stx and extend to other *E*. *coli* strains and other bacteria as well. SOS-induced mutation, also known as the mutator response or hypermutation, is a feature of the SOS response that can be induced by DNA–damaging agents in most or all *E*. *coli* strains studied [42, 43]. Since zinc inhibited other aspects of the SOS response, we thought zinc might inhibit the mutator response as well.

Although UV light, quinolone antibiotics and mitomycin C have been considered the classical drug activators of the SOS response, we found that many drugs used in clinical medicine are strong inducers of the SOS response, including drugs used for cancer chemotherapy such as 5-fluorouracil, arsenic trioxide, 6-mercaptopurine, and azacytidine. Nucleoside reverse transcriptase inhibitors (NRTIs) such as zidovudine and didanosine were also activators of the SOS response, as previously noted [13]. In addition, a chemical agent not classically included in this class, the herbicide paraquat, also induced the SOS response. Zinc acetate showed a strong inhibitory effect against zidovudine-induced SOS activation, just as it did against other activators such as ciprofloxacin, mitomycin C, trimethoprim, and H_2_O_2_ (Fig 1B and Ref. [4]). Of all the metals tested, only cobalt chloride showed SOS-inhibiting activity similar to zinc (Fig 1G). Cobalt is much more toxic to mammalian cells, however, than zinc. Indeed the toxicity of cobalt, as assessed by the U.S. Food and Nutrition Board, is 160 times that of zinc, based on the inverse ratio of the tolerable upper limit (TUL) of these metals; http://www.acu-cell.com/nico2.html. The zinc ionophore zinc pyrithione was about 80 times more potent than zinc acetate in inhibiting *recA* expression (Fig 1H), but enthusiasm for zinc pyrithione should also be tempered by its greater toxicity than zinc salts. Indeed, the concentration of zinc pyrithione effective at inhibiting *recA* activation, 10^−5^ M, or 10 μM, in Fig 1H is the same concentration at which toxicity was noted in human skin cells [44]. However, the increased potency of zinc pyrithione might be able to be exploited in specialized situations not relating to mammalian cells, such as in preventing emergence of resistance to anti-biofilm or anti-biofouling agents used on inanimate objects. In addition, we have shown that it is possible to achieve concentrations of zinc acetate as high as 0.4 mM in the lower GI tract of rabbits with high-dose oro-gastric administration [18], so perhaps it would not be necessary to resort to the more toxic zinc pyrithione.

We found we could observe SOS-induced hypermutation in many different *E*. *coli* strains of differing pathotypes, and also in *K*. *pneumoniae* and *E*. *cloacae*. The increase in the rate of mutation to rifampin resistance seemed to vary between strains, although we have not tested enough strains in each category to make state this with statistical certainty. Nevertheless, it seemed that the magnitude of the hypermutation response was greater in the two wild-type STEC strains (20- to 1000-fold increase compared to untreated controls) versus the other *E*. *coli* and Klebsiella strains (~ 10-fold increase). Several research groups have pointed out that STEC strains appear to be evolving quickly [45, 46], with changes even occurring during the course of infection in a single host animal or human [47, 48]. These predictions seemed to be borne out by the emergence of the STEC O104:H4 strain that caused the large outbreak in Germany in 2011 [49, 50]. Therefore, the data presented here on ciprofloxacin-induced mutation to rifampin resistance (Fig 2), to ß-glucuronidase-positivity (Fig 3), and to trimethoprim resistance (Fig 4) is consistent with the impression of other investigators that STEC may be more mutable than other *E*. *coli* strains.

Our experiments on the mechanisms of action of zinc in inhibiting the SOS response point toward an interaction with RecA itself. Our results in Table 2 clearly show that *recA* is absolutely required for the hypermutation response. While this result may seem predictable by those who are familiar with the SOS response, clinicians interested in antibiotic resistance might be surprised to see that a single gene, *recA*, can affect hypermutation so strongly. Our course, other loci such as *lexA*, mismatch repair genes, *dinB*, and *umuDC* also play important roles in SOS-induced mutation. Our experiments in Fig 5 show that zinc blocks the cleavage of LexA both in living cells as well as in an *in vitro* LexA cleavage assay. As mentioned above, the apparent interaction between zinc and the ATP analog (Fig 5F) might indicate that zinc interferes with RecA’s ATP binding site, which is essential for RecA activation [51]. We are currently testing if zinc changes the ability of RecA to bind to ssDNA by electrophoretic mobility shift assays (EMSAs), or blocks the ability of RecA to hydrolyze ATP to ADP, as occurs when RecA dissociates from the ssDNA. Neither RecA nor LexA contains a canonical zinc-binding domain, such as a zinc finger motif or histidine-rich region. But this is not really a surprise, because these domains have such a high affinity for zinc that they remain replete with zinc under most physiological conditions, whereas the inhibitory effects of zinc on the SOS are only observed at stressfully high concentrations of zinc, suggesting that the zinc binding site is of lower affinity, and therefore only occupied in the presence of high zinc.

RecA was identified several years ago as a target for drug development with a goal of developing resistance inhibitors [52–54]. Although small molecule inhibitors were identified that could block RecA activity in vitro in broken-cell assays, these inhibitors were frequently unable to cross the Gram-negative cell wall, were thus inactive against live bacteria, and so enthusiasm for this line of research waned. Zinc and zinc-containing compounds could revive the search for effective and cell-permeant SOS inhibitors in *E*. *coli* and other Gram-negative bacteria.

While looking ahead to being able to define the exact site of action of zinc, we also look ahead to the possibility of one day harnessing the SOS-inhibiting properties of zinc to limit the emergence of antibiotic resistance in clinical medicine. Low concentrations of antibiotics, similar to those used to induce hypermutation in the experiments described here, can persist in the human GI tract for days to weeks after the completion of a course of therapy, in the GI tract of animals fed low doses of antibiotics for growth promotion, and in sewage. Patients receiving anti-retroviral therapy for HIV or cancer chemotherapy are also being inadvertently exposed to drugs with strong SOS-inducing abilities, thus paving the way for emergence of antibiotic resistance from their endogenous microbiota, even before they have received a single dose of an actual antibiotic. Although many hospitals are now struggling to implement meaningful antibiotic stewardship programs for antibacterial drugs, in the future antibiotic stewardship may have to broaden even further to include safer, smarter use of SOS-inducing drugs.

Although zinc salts appear to have promising ability to prevent the SOS response and block the mutator response, in the antibiotic resistance field one must think a few steps ahead, as in a chess match, to consider the possible counter-moves of one’s opponent. In this context we must consider whether *E*. *coli* or other bacteria could develop resistance to zinc or other hypothetical resistance inhibitors. In general, bacteria have been much slower to develop resistance to metals than to other antibacterial compounds, but some exceptions have been noted [55]. In the future, solving the problem of antibiotic resistance may require a combination of drugs, including virulence inhibitors and resistance inhibitors, used in clever ways so as to achieve “synthetic lethality” or “selection inversion”[56]. Including zinc in these strategies would help galvanize research progress toward these goals [57].

## Supporting information

S1 Fig Ability of Ciprofloxacin to Induce Mutation to Rifampin at 100 mg/L, or 10 times the MIC, in strain JLM281. Panel A, Rifampin resistance frequency as a function of ciprofloxacin concentration. Panel B, Rif ^R^ colonies of JLM281 on LB + 100 mg/L rifampin, showing normal or near-normal colony size. Panel C, ciprofloxacin-induced mutation to rifampin resistance (at 100 mg/L rifampin) was inhibited by zinc acetate, as shown for other strains in Fig 2.
(TIFF)

S2 Fig A trivial explanation for the findings in Fig 2 would be if zinc acetate increased the MIC's for ciprofloxacin on the strains we tested, thus reducing the intensity of the SOS response induced by the ciprofloxacin. Alternatively, if zinc decreased the rifampin MIC of the strains, fewer rifampin-resistant colonies would be observed in the presence of zinc, leading to the false conclusion that zinc was inhibiting hypermutation. To rule out these trivial explanations, we measured the MICs of ciprofloxacin and rifampin using the E-test method. We inoculated our plates with bacteria at a dilution of 1: 100 from overnight. Panel A, photograph of STEC Popeye-1 grown on DMEM plates with varying concentrations of zinc acetate, from zero to 0.2 mM. Panel B, graph of ciprofloxacin MICs vs. zinc for Popeye-1. The slight decrease in ciprofloxacin MIC observed refuted the trivial explanation as an explanation for our findings. Similarly, we tested the effect of zinc on the rifampin MIC of several strains. Panel C, lack of effect of zinc on the rifampin MIC for strains Popeye-1, CP9, and B171-8. Panel D, photograph showing that the rifampin MIC remained unchanged in the presence of zinc for strain B171-8. In Panel D, the concentration of added zinc was, from left to right, zero, 0.05, 0.1, and 0.2 mM. As shown in this figure, zinc acetate at concentrations up to 0.2 mM had no effect on the rifampin MIC on LB (Panels C and D).
(TIF)

## Abbreviations

Stx: Shiga toxin
STEC: Shiga-toxigenic *Escherichia coli*
EPEC: enteropathogenic *Escherichia coli*
ExPEc: extra-intestinal pathogenic *E*. *coli*
SOS: “Save Our Ship”
MUG: methyl-umbelliferyl-ß-D-glucuronide
