# Supplementary figures and images for: Zinc blocks SOS-induced antibiotic resistance via inhibition of RecA in Escherichia coli
Source: PLoS One. 2017 May 22;12(5):e0178303. doi: 10.1371/journal.pone.0178303 (PMC5440055; doi:10.1371/journal.pone.0178303)

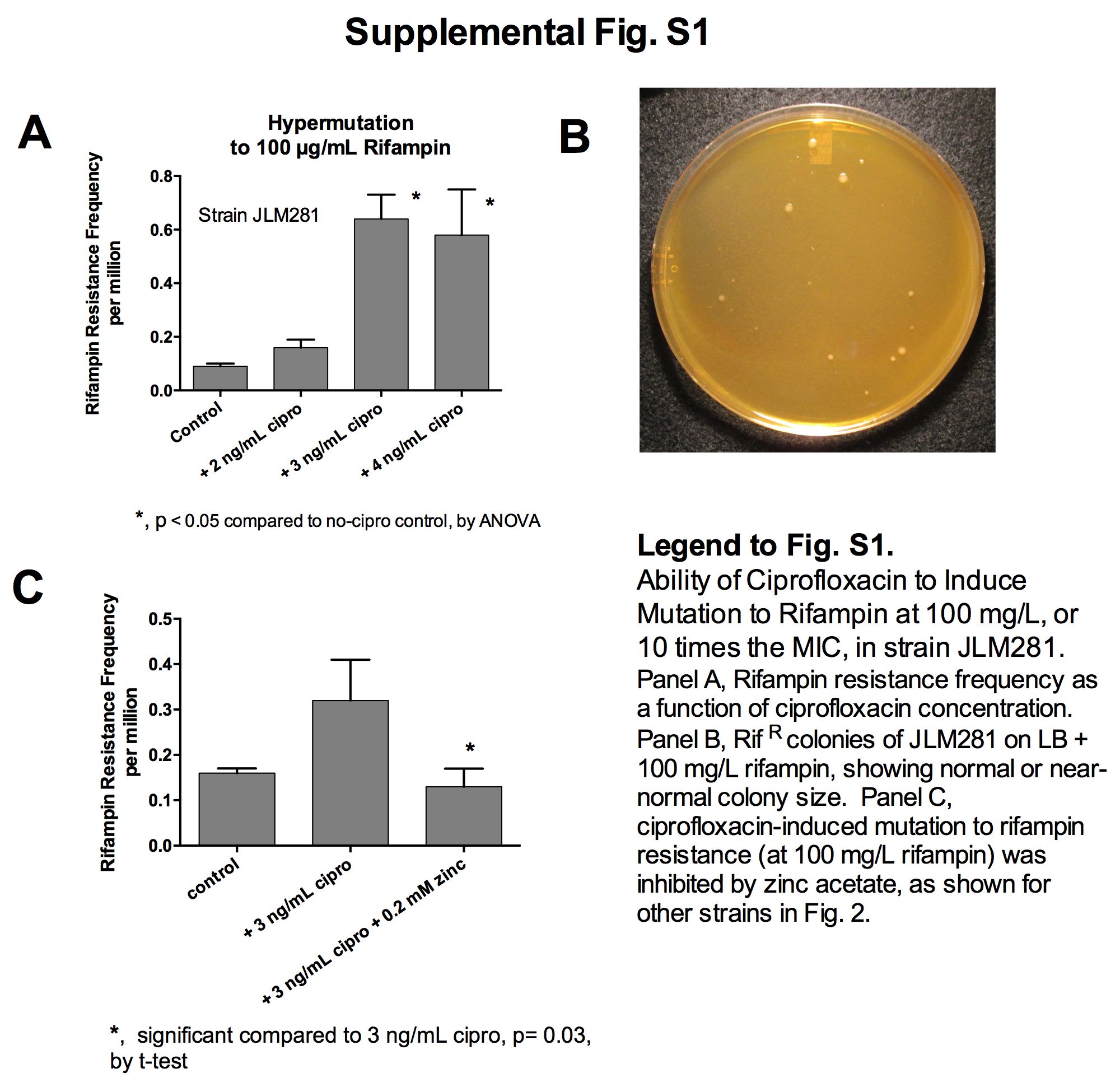

Supplement: S1 Fig — Panel A, Rifampin resistance frequency as a function of ciprofloxacin concentration. Panel B, Rif R colonies of JLM281 on LB + 100 mg/L rifampin, showing normal or near-normal colony size. Panel C, ciprofloxacin-induced mutation to rifampin resistance (at 100 mg/L rifampin) was inhibited by zinc acetate, as shown for other strains in Fig 2. (TIFF) [file pone.0178303.s001.tiff]
